# Implementing the DEcision-Aid for Lupus (IDEAL): study protocol of a multi-site implementation trial with observational, case study design: Implementing the DEcision-Aid for Lupus

**DOI:** 10.1186/s43058-021-00118-9

**Published:** 2021-03-11

**Authors:** Jasvinder A. Singh, Larry R. Hearld, Allyson G. Hall, T. Mark Beasley

**Affiliations:** 1 Medicine Service, VA Medical Center, 700 19th St S, AL Birmingham, 35233 USA; 2 Department of Medicine at School of Medicine, University of Alabama at Birmingham, 1720 Second Ave South, Birmingham, AL 35294-0022 USA; 3 Division of Epidemiology at School of Public Health, University of Alabama at Birmingham, 1720 Second Ave South, Birmingham, AL 35294-0022 USA; 4 University of Alabama at Birmingham, Faculty Office Tower 805B, 510 20th Street S, Birmingham, AL 35294-0022 USA; 5 Department of Biostatistics, University of Alabama at Birmingham; Birmingham/Atlanta VA Geriatric Research, Education, & Clinical Center, Department of Veteran’s Affairs, 510 20th Street S, Birmingham, AL 35294-0022 USA

**Keywords:** Decision-aid, Lupus, Systematic lupus erythematosus, Lupus, Implementation, Trial

## Abstract

**Objective:**

To provide the details of the study protocol for an observational, case study design, implementation trial.

**Methods:**

Implementing the DEcision-Aid for Lupus (IDEAL) study will put into practice a shared decision-making (SDM) strategy, using an individualized, culturally appropriate computerized decision-aid (DA) for lupus patients in 15 geographically diverse clinics in the USA. The overarching frameworks that guide this implementation study are the Consolidated Framework for Implementation Research (CFIR) and Powell’s typology of implementation strategies. All 15 clinics will receive standardized capacity-building activities for lupus DA implementation in the clinic, including education, training, technical assistance, re-training, and incorporation of a clinic champion in the core team of each site. In addition, clinics will also choose among c*linic-targeted activities* to integrate the DA into existing work processes and/or *patient-targeted activities* to raise awareness and educate patients about the DA. These activities will be chosen to stimulate participant recruitment and retention activities that support the implementation of the DA at their clinic. In study aim 1, using surveys and semi-structured interviews with clinic personnel in 15 lupus clinics, we will assess stakeholder needs and identify clinic and contextual characteristics that inform the implementation strategy component selection and influence implementation effectiveness*. Study aim 2* is to implement and assess the effectiveness of the IDEAL (standardized and tailored) strategy in 15 lupus clinics by examining the changes in our primary outcome of penetration, i.e., the proportion of all eligible patients in the clinic that receive the lupus DA, and secondary outcomes include DA appropriateness, acceptability, success, permanence, and feasibility. *Study aim 3* is to identify ways to sustain and disseminate our lupus DA via semi-structured debriefing interviews with key clinic personnel and patients.

**Discussion:**

The study will enroll at least 500 patient participants with lupus across all 15 sites and assess the effectiveness in implementing the DA in various clinic settings across the USA.

**Trial registration:**

ClinicalTrials.gov, NCT03735238. Protocol version number: 15, date 6/8/2020

**Supplementary Information:**

The online version contains supplementary material available at 10.1186/s43058-021-00118-9.

Contributions to the literature

- Our study provides insights into a contextually driven approach to assessing implementation strategies that allows participants to choose from a menu of strategies that are believed to be most effective for their unique circumstances (e.g., geography, resources).
- We leverage multi-method, longitudinal data over a 3-year period that enable us to understand how the implementation strategies and their effects on implementation outcomes change over time and why.
- Given the study period encompasses the COVID-19 outbreak, our study will shed light on how clinics and researchers can adapt to major exogenous events to implement and evaluate evidence-based interventions.

## Introduction

Systemic lupus erythematosus (SLE) is a multi-system autoimmune disease that typically affects young women. SLE, commonly referred to as lupus, is a rare disease that affects 0.1% of the general population but causes 2% of the end-stage kidney disease in the USA [1]. Decision-making for the use of medications is frequently poor. Many lupus patients decline life-saving immunosuppressive medications for their treatment [2–5], at least partly due to the lack of recognition of associated benefits and a fear of side effects [6–10]. Significant health disparities in outcome in lupus have been described [11–14], with 2.4 times higher mortality than age-matched controls [15–18].

In a PCORI-funded, multicenter randomized trial, 301 high-risk adult women with lupus kidney disease, including racial/ethnic minorities with low socio-economic status, either received an individualized, evidence-based, patient-centered, computerized lupus decision-aid (DA) [19–24], or the American College of Rheumatology (ACR) lupus paper pamphlet with information on treatments [25]. Compared to the ACR paper pamphlet, people who used the lupus DA had a much greater decrease in decisional conflict (uncertainty in choosing options) for immunosuppressive drugs and were more likely to choose the treatment option most consistent with their values, having viewed information that mattered the most for the treatment decision [26]. Compared to the pamphlet group, more patients rated the information in lupus DA to be excellent for understanding the impact of lupus (49% vs. 33%), risk factors (43% vs. 27%), medication options (50% vs. 33%), and evidence about medications (47% vs. 24%) and rated the ease of use of materials higher (51% vs. 38%; *p* values ≤ 0.006 for all) [26]. The RCT demonstrated the effectiveness of the lupus DA [26]. However, implementation of effective interventions is difficult and does not always succeed. Therefore, the focus needs to be on the implementation in order to ensure the lupus DA reaches as many people as possible.

The Implementing the DEcision-Aid for Lupus (IDEAL) study will use an observational case study design to evaluate the effectiveness of different implementation strategies to implement this computerized decision-aid (DA) for lupus patients in 15 clinics located across the USA, using an observational case study design.

## Methods

### Study setting

We strategically chose 15 US clinics from diverse settings to permit evaluation of success (and failure) in different contexts. Clinics are diverse with regard to the location (urban vs. suburban), region of the country, number of patients seen, type of clinic (general rheumatology vs. lupus vs. rheumatology/renal clinic), and practice type (private vs. academic vs. private/academic hybrid; Table 1). The study is registered at ClinicalTrials.gov (ID: NCT03735238).

**Table 1:** Participating sites, practice type, clinic type (general rheum, lupus), location, # lupus patients, site PIs, and region

Name	Practice	Type of clinic	Location	# lupus pts	Site PI	Region
1. Washington U, St. Louis	Private	General rheum	Suburban	> 200	A. Kim	Mid-west
2. Ohio State U, Columbus	Academic	Rheum/renal	Urban	185	A. Meara	Northeast
3. Loyola University, Chicago	Private	General rheum	Suburban	500	Z. Aouhab	Mid-west
4. Vanderbilt U, Nashville	Academic	Lupus/renal	Urban	> 400	N. Annapureddy	South
5. UCLA, Los Angeles	Academic	General rheum	Urban	400–600	M. McMahon	West
6. Medical U South Carolina, Charleston	Academic	Lupus	Urban	> 1000	D. Kamen	South
7. Baylor University, Houston	Academic	Lupus	Urban	300	K. Bhairavarasu	South
8. Emory University, Atlanta	Academic	Lupus	Urban	> 300	S. Lim	South
9. Northwestern University, Chicago	Academic	General rheum	Urban	200–250	R. Ramsey-Goldman	Mid-west
10. Northwell Health, Long Island, NY	Private	General rheum	Suburban	500–600	S. Narain	Northeast
11. U of Mississippi, Jackson	Academic	General rheum	Urban	300–350	V. Majithia	South
12. UCSD, San Diego	Academic	Lupus	Urban	550	K. Kalunian	West
13. U of Alabama at Birmingham (UAB)	Hybrid	Lupus	Urban	700	W. Chatham	South
14. Cedars-Sinai Hospital, Los Angeles	Private	General rheum	Suburban	500	S. Venuturupalli	West
15. U of Chicago, Chicago	Academic	General rheum	Urban	200–300	K. Trotter	Mid-west

### Study-specific aims, study eligibility criteria, and the conceptual model

**Fig. 1:**
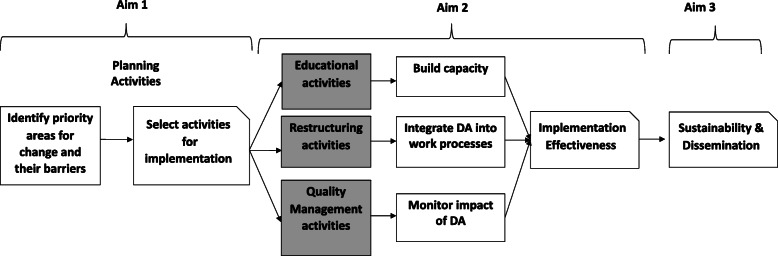
Conceptual and evaluation logic model. Boxes with snip corners indicate the outcome of each aim; empty boxes without color indicate intermediate steps to achieve aims; aim 2 activities are in the grey colored boxes

Study-specific aims and study inclusion and exclusion criteria are summarized in Table 2. The overarching frameworks that guide this implementation study are the Consolidated Framework for Implementation Research (CFIR) [27] and Powell’s typology of implementation strategies [28] (Fig. 1). The CFIR organizes the factors that influence implementation into five domains, which will guide the semi-structured interview protocols for aims 1 and 3 (Fig. 1). Aim 2 will use Powell’s typology to thematically group implementation activities.

**Table 2:** Study-specific aims and study inclusion and exclusion criteria

Specific aim	Inclusion criteria	Exclusion criteria
Specific aim 1: To assess stakeholder needs and identify clinic and contextual characteristics (e.g., readiness for change, physician attitudes, patient barriers) that inform IDEAL strategy component selection and influence implementation effectiveness through the conduct of a formative evaluation in 15 lupus clinics		
Specific aim 1 clinic personnel	Clinic personnel involved in the care processes of lupus patients	Clinic personnel not involved in the care processes of lupus patients
Specific aim 2: To implement and assess the effectiveness of the IDEAL (standardized and tailored) strategy in 15 lupus clinics by examining changes in subjective and objective measures of implementation effectiveness over 27 months through an observational, case study design where each clinic serves as a case		
Specific aim 2 patients	Men and women ≥ 18 years of age of all races/ethnicities with a diagnosis of systemic lupus erythematosus, regardless of current active flare	No diagnosis of lupus; not English or Spanish speaking; visually impaired; or have altered mental status
Specific aim 2 clinic personnel	Clinic personnel involved in the care processes of lupus patients	Clinic personnel not involved in the care processes of lupus patients
Specific aim 3: To identify opportunities for sustaining and disseminating our lupus DA via semi-structured debriefing interviews with key clinic informants of aim 1 and patients of aim 2 and develop a manual that provides a step-by-step implementation guide for incorporating lupus DA into regular clinic visits and care pathways for lupus patients		
Specific aim 3 clinic personnel	Participating clinical personnel from specific aim 1	Clinic personnel who are not involved in specific aim 1
Specific aim 3 patients	2 patient participants randomly chosen from patient participants from specific aim 2 from each of the 15 clinic sites	Patients with lupus who are not involved in specific aim 2

### Interventions

All 15 clinics will receive standardized capacity-building activities prior to starting lupus DA implementation in the clinic, including the following (Fig. 1):

- Education: a series of 60-min seminars aimed at all 15 clinics that educates clinic personnel about the decision-aid including its purpose, content, and supporting evidence of effectiveness
- Implementation coaching: a series of webinars, offered 1–2 months after each sites’ formative evaluation, that describes the clinic-specific findings of the formative evaluation, reviews the implementation strategies available to the clinic (both standardized and tailored), and jointly identifies the preferred strategies for the clinic
- Technical assistance: ongoing, ad hoc technical support on the use and maintenance of an electronic tablet (e.g., trouble launching the DA, problems navigating screens)
- Clinic champion: designated member of the clinic who is dedicated to supporting the implementation of the DA in the clinic
- Refresher training course: a webinar conducted every 6 months or as needed that describes the implementation strategies and common barriers being confronted across the participating clinics and shares best practices of how these strategies are being deployed

In addition, clinics will choose to engage in either or both of the following two activities designed to stimulate DA implementation in their clinic: (1) *clinic-targeted activities* to further integrate the DA into existing work processes and (2) *patient-targeted activities* to raise awareness and educate patients about the DA (Table 3). At the end of the study, all sites will be reviewed to determine which strategies were used throughout the study (Fig. 1).

**Table 3:** Implementation strategy component, based on barriers and facilitators from our pilot work with target patient population and clinics

Component	Barriers (B) and facilitators (F) from pilot study addressed through implementation strategy
**Standardized capacity-building activities**	
Education, training, technical assistance	Training staff on iPad and DA use (F); keeping iPads charged (B); IRB concerns (B)
Clinic champion	Staff time shortage/buy-in (B); convincing multiple providers (B); change fatigue (B)
Refresher training course	Knowledge retention (F)
**Tailored, clinic-targeted activities**	
DA reminder in patient intake process	Coordinating/training front desk staff (F); slowing clinic flow (B)
Audit and feedback	Engaging clinic staff and maintaining engagement (F); training front desk staff (F)
Team huddles/clinic meetings	Staff time shortage/buy-in (B); failure to systematically apply DA into existing processes (B)
**Tailored, patient-targeted activities**	
Pre-visit patient portal message with DA link	Length of the DA (B); need to complete other surveys during the visit (B)
Paper-based version of the lupus DA	Too much information at once (B); patient mood, language, background, and understanding (B)
Clinic poster about the lupus DA	Understanding/interest (B)
DA information via waiting room TV/kiosk	Language/literacy/understanding/interest (B)

### Outcomes

#### Primary outcome measure

1. Penetration: this is measured using study records and/or electronic health records (# of patients who viewed the DA/# of eligible patients) [time frame 24 months].

#### Secondary outcome measures

1. Perceived acceptability of intervention measure (AIM): clinic personnel’s perception of the acceptability of the decision-aid, measured using a validated scale with four (4) items with responses ranging from 1 (“completely disagree”) to 5 (“completely agree”). The four items will be averaged to create one composite mean scale score (range 1–5), where higher scores reflect perceptions of greater acceptability (i.e., better outcome) [time frame 12 months].
2. Perceived DA implementation success: clinic personnel’s perception of the implementation success of the decision-aid, measured using a validated scale with three (3) items with responses ranging from 1 (“disagree”) to 5 (“agree”). These three items will be averaged to create one composite mean scale score (range 1–5), where higher scores reflect perceptions of greater implementation success (i.e., better outcome) [time frame: 12 months].
3. Perceived DA permanence: clinic personnel’s perception of the permanence of the decision-aid, measured using one validated item that is scored ranging from 1 (“not at all permanent”) to 5 (“extremely permanent”). This item will be examined by itself, where higher scores indicate perceptions of greater permanence of the decision-aid in the clinic (i.e., better outcome) [time frame 12 months].
4. Patient perception of DA usefulness: patient perception of the effect of the decision-aid on preparing the patient for decision-making measured using the preparation for decision-making (PDM), a validated scale consisting of 10 questions scored on an ordinal scale from not at all = 1 to a great deal = 5. For scoring, sum the score of the 10 items and divide by 10. Scores can then be converted to a 0–100 scale by subtracting 1 from this summed score and multiplying by 25 [time frame 0 months (baseline)].
5. Patient satisfaction with DA: patient satisfaction with the ease of the use of the decision-aid measured using a validated single-item scale scored on an ordinal scale from strongly disagree = 1 to strongly agree = 5. This item will be examined by itself, where higher scores indicate greater patient satisfaction with the decision-aid (i.e., better outcome). This is a single-item scale, and there are no subscales. It was adapted from another study that assessed satisfaction with iPad or interactive voice response [time frame 0 months (baseline)].
6. Perceived intervention appropriateness measure (IAM): clinic personnel’s perception of the appropriateness of the decision-aid, measured using a validated scale with four (4) items with responses ranging from 1 (“completely disagree”) to 5 (“completely agree”). The four items will be averaged to create one composite mean scale score (range 1–5), where higher scores reflect perceptions of greater appropriateness (i.e., better outcome). [time frame 12 months].
7. Perceived feasibility of intervention measure (FIM): clinic personnel’s perception of the feasibility of the decision-aid, measured using a validated scale with four (4) items with responses ranging from 1 (“completely disagree”) to 5 (“completely agree”). The four items will be averaged to create one composite mean scale score (range 1–5), where higher scores reflect perceptions of greater feasibility (i.e., better outcome) [time frame 12 months].

Study outcomes that will be assessed at the level of research team, clinic/practice personnel and patients are shown in Table 4. These include #activities pursued at each clinic, change in penetration, decision-aid acceptability, appropriateness and feasibility [29, 30], decision-aid success [31], and decision-aid permanence [32]. Patient-centered outcomes include decisional conflict [33–38], patient-physician care process [39], patient involvement in decision-making [40–43], patient acceptability [44] and feasibility [45, 46], decision-aid review time and patient satisfaction [45], patient perception of decision-aid usefulness [47] and patient-physician communication [48] (Table 4).

**Table 4:**
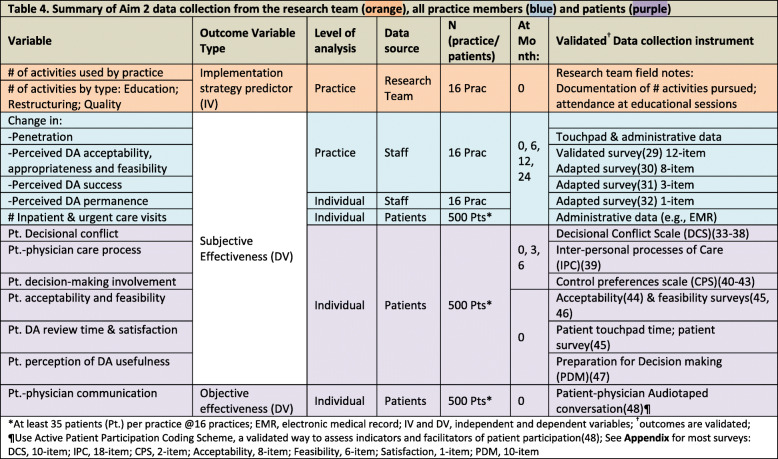
Summary of Aim 2 data collection from the research team (orange), all practice members (blue) and patients (purple)

^a^At least 35 patients (Pt.) per practice @16 practices; EMR, electronic medical record; IV and DV, independent and dependent variables; DA, decision-aid

^b^outcomes are validated; ^c^Use Active Patient Participation Coding Scheme, a validated way to assess indicators and facilitators of patient participation [48]; DCS, 10-item; IPC, 18-item; CPS, 2-item; Acceptability, 8-item; Feasibility, 6-item; Satisfaction, 1-item; PDM, 10-item

### Participant timeline

**Fig. 2:**
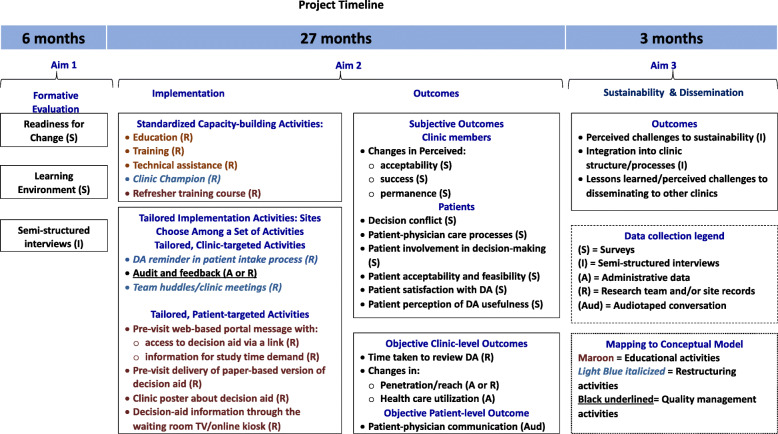
Summary of study-specific aims and project timeline. Each site will get the capacity-building strategy and chose among a set of clinic-targeted or patient-targeted activities, which map to our conceptual model

The project and participant timeline is shown in Fig. 2. The participant timeline for aim 2 is summarized in Table 4.

### Sample size

No sample size calculations were done for specific aims 1 and 3, which were convenience samples and done for descriptive purposes. We targeted at least 4 semi-structured phone interviews and surveys per site for aim 1 (average 6–8/site); repeat for aim 1 duirng the study follow-up, and in addition interview 2 patients per site.

A power analysis for the quantitative portion of specific aim 2 was based on the following assumptions for a sample size of 500 participants: (1) 15 clinics and (2) an intraclass correlation coefficient of 0.05. This provided us with a total confidence interval width of 5.3% for 10% of the total clinic population that is administered the DA to a width of 8.1%, if 40% of patients are administered the DA. For outcomes on a 5-point Likert scale, the corresponding 95% CI widths for a standard deviation of 0.5 and 2.0 on the mean score is estimated to be 0.09 and 0.35, respectively. For individual-level outcomes measured as a rate (e.g., # visits/1000) or as means (e.g., Likert scale), we anticipate small margins of error. A minimum number of proposed sites (*n* = 15) are needed to comprehensively assess how different implementation strategies are influenced by and interact with different contextual conditions (different geographic areas, clinic type, and patient diversity) to affect implementation effectiveness and, thus, determine the generalizability of the study findings. However, given the proven effectiveness of the DA, and in the spirit of implementation research, we will continue to recruit to our proposed maximum sample size target of 4000.

### Recruitment and retention

#### Key clinic personnel recruitment and retention

Semi-structured phone interviews with 6–8 key clinic personnel. Clinic personnel surveys of up to 15 clinic personnel per site. End of study semi-structured phone interviews with the same 6–8 key clinic personnel as in aim 1, and 2 patients per site selected by the PI.

#### Patient participant recruitment and retention

We will recruit and enroll up to 4000 adults (≥ 18 years of age; from the 15 sites) who have been diagnosed with lupus during a regular clinic visit. Adult men and women of all races/ethnicities with lupus will be recruited by the study coordinator/site PI. Paper flyer advertisements with study information will be placed in clinics and waiting rooms. Other recruitment strategies will be implemented at some sites, such as DA information offered on the patient portal, a paper-based version of the DA mailed to the patient, clinic posters, and/or study information through waiting room TV or online kiosk.

### Data collection methods

This study will be coordinated by the University of Alabama at Birmingham (UAB). All study visits and procedures will be performed at the 15 sites. Enrollment will be competitive. Participants will be seen according to the details that follow:

#### Specific aim 1: Clinic personnel survey and semi-structured interviews

Clinic personnel will be emailed a link to an online Qualtrics® survey. An automatic email will be generated in the Qualtrics® system and will go out to all non-responders every 7–8 days.

A 45 to 60-min semi-structured phone interview will assess stakeholder needs and identify clinic characteristics (e.g., policy environment, physician, clinic, patient barriers) that influence DA implementation and choice of the implementation strategy. The interview protocol will be adapted to fit different key personnel. A link to the brief video animation introducing the decision-aid use and content along with the interview protocol and copy of the decision-aid will be emailed to clinic personnel prior to being interviewed. All interviews will be recorded and transcribed verbatim for accuracy.

#### Specific aim 2: Implementation

##### Patient baseline visit

The baseline visit will take approximately 1 h to complete. All adults with lupus, who understand English or Spanish, are eligible. Once the potential participant checks in either at a kiosk or with a front desk clerk at their regularly scheduled lupus clinic appointments, they will be made aware of the lupus DA by either the clinic clerk or the study coordinator. After informed consent and privacy assurance, a participant number will be assigned. During the baseline visit, the study coordinator will assess the inclusion/exclusion criteria.

Participants will watch a short informational video on an electronic tablet, review the computerized lupus DA, and complete the questionnaire on Qualtrics® using an electronic tablet. Each patient participant will be reminded of the 3- and 6-month follow-up phone calls prior to the visit departure.

##### Specific aim 2: Follow-up patient participants

At 3 and 6 months post baseline visit, patient participants will be administered a 15-min survey via phone, email, or phone text with a link. Patient participants will receive a $10 payment for completion. Follow-up reminders will be done for any/all methods. Non-responders will be administered the survey on their next regularly scheduled lupus clinic visit. If requested, surveys will be mailed to patient participants via US mail and will include a pre-stamped return envelope.

##### Specific aim 2: Clinic personnel survey

Up to 15 clinic personnel, the same clinic personnel as surveyed in aim 1, will be emailed a link to complete an online Qualtrics® survey at 0, 6, 12, and 24 months to assess their perception of appropriateness, acceptability, success, permanence, and feasibility of the decision-aid. This survey will take between 10 and 15 min to complete. A reminder email will be sent to all non-responders every 7–8 days thrice.

#### Specific aim 3: Semi-structured interviews

Clinic personnel interviewed for specific aim 1, as well as 2 participants at each site (selected by the site PI), will be administered a 45–60-min semi-structured phone interview. This interview will identify opportunities for sustaining and disseminating a lupus DA and help to develop a manual that provides a step-by-step implementation guide for incorporating lupus DA into regular clinical visits.

### Data management

Dr. Beasley, lead statistician, will supervise and oversee all quantitative data management at the UAB IDEAL Data Coordinating Center (DCC) and analysis for IDEAL DCC. Questionnaires will be tested before being uploaded into Qualtrics®. Data entered into Qualtrics® by participants will be captured into the IDEAL electronic Data Entry System (eDES; Birmingham, AL) directly using iPads for seamless data management and auditing across the IDEAL sites. The DCC will ensure that the data collected are of the highest quality possible and will be accomplished in part by having thorough edit checks as close to the collection in time as possible, and updated as needed to guarantee high-quality data through quality control and quality assurance. Edit checks will be reviewed by Dr. Beasley on an ongoing basis to evaluate whether any checks need to be added or any existing checks need to be modified. All quantitative analyses will be conducted using SAS (Cary, NC) version 9.4 or higher or R-routines for specialty programs as needed. All qualitative analyses will be conducted with NVivo 12, available through the UAB School of Medicine.

### Statistical methods

#### Specific aim 1

The purpose is to identify and quantify clinic and contextual characteristics (e.g., readiness for change, physician attitudes, patient barriers) to inform strategy component selection and influence implementation effectiveness of lupus DA to be evaluated in specific aim 2. Thus, aim 1 is largely descriptive and qualitative, and there are no formal statistical hypotheses (Additional file 1: Appendix 1). Overall rates and 95% confidence intervals will be computed. We will also perform qualitative analyses of semi-structured interview data by first coding the data using the major domains of the Consolidated Framework for Implementation Research (CFIR) as codes (innovation characteristics, individual characteristics, inner setting, outer setting, process), investigators discussing differences in domain definitions, reconciling, and then analyzing for key themes related to how readiness to implement change varied as a function of clinic characteristics, i.e., we will focus on themes pertaining to the inner setting.

#### Specific aim 2

For individual-level outcomes, initial analyses will focus on how clinic personnel and patient perceptions change over time. The statistical null hypothesis is the assumption that there is no change over time vs. the alternative that there is a change over time. For the analytic approach, we will examine the mean levels (total change) and quantify with means (or proportions, as appropriate) and 95% confidence intervals. We will also examine the patterns of change (growth trajectories) while controlling for differences between clinics using multivariable models that account for repeated measures and clinic clustering of patients (i.e., generalized estimating equations (GEE) with robust standard errors). Additional analyses may examine by-group comparisons of clinics determined by clinical characteristics (e.g., geographic areas, clinic type, and patient diversity) observed in specific aim 1. Any such by-group analyses will be conducted separately. A primary independent variable of interest is the type of strategy used for implementation. Following the completion of the study, each sites’ strategies will be evaluated, and sites will be grouped accordingly. A covariate allowing for testing group differences will be included in the multivariable models to assess whether trajectories differ by implementation strategy.

The second component of our evaluation of specific aim 2 will use fuzzy set qualitative comparative analysis (fsQCA) [49, 50] to assess the effectiveness of implementation strategies for clinic-level outcomes. For patient-physician communication analysis, question-asking, assertive responses, and expressions of concern will be coded as patient participation; supportive talk and partnership building by provider will be coded as active physician communication. Other outcomes will be obtained from the electronic health records, including healthcare utilization outcomes (e.g., inpatient visits and ER/urgent care visits) and, missed/canceled appointments.

#### Specific aim 3

The analytic approach is largely descriptive, and there are no statistical hypotheses. Specific qualitative analyses are described in Additional file 1: Appendix 1. Qualitative methods will be similar to those described above for aim 1, except that the focus will be to identify opportunities for sustaining and disseminating a lupus DA.

### Data monitoring

This study poses no more than minimal risk to participants, and therefore, there will be no Data Safety Monitoring Board (DSMB). No clinical monitoring will be required for this study.

Quality control (QC) procedures have been implemented beginning with the Qualtrics® data entry system and data QC checks that will be run on the database once enrollment begins. All personal identifiers collected will be stored on a secure UAB server and backed up nightly. Survey data will be merged with baseline enrollment data to create master SAS datasets. Logic and range checks in SAS 9.4 (Cary, NC) will ensure the identification of data accuracy (see section 9.4). Any missing data or data anomalies will be communicated to the site(s) for clarification/resolution.

### Potential Benefits and Harms

A potential benefit from this study is that the knowledge gained will provide patients valuable information about lupus and its treatment, as well as to inform clinicians about the best way to implement the decision-aid into normal clinic workflow. We will take the following precautions to avoid/minimize risks to participants.

We will make it a top priority to protect patient privacy, confidentiality, and autonomy for all study procedures, summarized as follows. Patient participant initial data and responses to questionnaires will be captured during regularly scheduled lupus clinic visits in a database behind the UAB firewall. Follow-up surveys will be conducted via phone, email, or in the clinic visit depending on the preference of the patient. All clinic personnel surveys and interviews will be conducted during general work hours (even before or after their shift). All semi-structured in-depth interviews will be captured via phone and transcribed for accuracy as soon as possible. All data (except data from semi-structured interviews) will be entered into coded electronic case report forms (CRF) and will be checked by study personnel daily for accuracy. On a quarterly basis, study investigators and the research team will review GCP, human subject’s protections/confidentiality, and study procedures. The research team will meet biweekly to review recruitment, enrollment, source documents, and electronic case report forms; in the event an adverse event occurs, this will be reported to the UAB IRB at the time of continuing review. All serious adverse events (SAEs) will be reported to the IRB and PCORI within 48 h of the principal investigator becoming aware of the event. All research team members will be informed by the PIs about any unanticipated problems involving risks to the participant. If any protocol changes are needed, the PIs will submit a modification request to the IRB. Protocol changes will not be implemented prior to IRB approval unless necessary to eliminate apparent immediate hazards to the research participant. In such cases, the IRB will be promptly informed of the change following implementation (within 1 week).

Considering the aim of this study is to determine the best practices for implementation of a treatment decision-aid, it poses no more than minimal risk to participants. We will use a secured database to minimize the risk of disclosure of personal health identifiers (PHI); therefore, no Data Safety Monitoring Board (DSMB) will be convened.

### Auditing

There is no formal auditing planned for this study.

### Research ethics approval

No study procedures will be performed before the approval of the institutional review board (IRB) at the University of Alabama at Birmingham (UAB) and at all other clinical sites. Each site will also perform the continuing annual review of the study. A copy of site ethics committee approvals and the approval of protocol amendments will be stored at each site. Copies of the ethics committee approvals for the UAB coordinating center will be provided to each site, and additional approvals will be obtained at the site as needed.

### Protocol amendments

All protocol amendments for the UAB coordinating center will be provided to each site promptly. These will also be discussed and reviewed (as needed) during the monthly project calls with each site.

### Consent

A non-signature consent form will be provided to all participants (both clinic personnel and patient participants), as permitted by Institutional Review Boards. The non-signature consent will describe the study in detail and payment procedures, as well as give participants important contact information for study personnel. Participants will be informed that participation is voluntary and that they may withdraw from the study at any time. Study coordinators will review the consent with the patient and answer any questions that may arise. Limited personal health identifiers (PHI) may be used (audio recording with physician, etc.), so all patient participants will be asked to sign a HIPPA form and will be given a copy for their records and a copy will be kept in the study binder. The reason for non-participation will be recorded. Some study sites will use consent forms with patient's signature, as required by their Institutional Review Boards.

Clinic staff participating in the study will be informed about the study by their nurse manager/supervisor and that participation is voluntary and they may withdraw from the study at any time. Staff will have the opportunity to carefully review the information sheet and HIPPA form prior to signing the HIPPA document. Clinic staff participating in the clinic survey and the semi-structured interviews will be given the information sheet and HIPPA form. A copy of the information sheet and HIPPA form will be given to all staff for their records.

### Confidentiality

Participant confidentiality and privacy will be strictly held in trust by the participating investigators, their staff, and their interventions. Therefore, the study protocol, documentation, data, and all other information generated will be held in strict confidence. All research activities will be conducted in as private a setting as possible. The information obtained during the conduct of this study is confidential, and disclosure to third parties other than those noted below is prohibited. The results of the research study may be published, but study participant’s names or identities will not be revealed.

### Declaration of interests

Each academic institution’s conflict of interest review board has established policies and procedures for all study group members to disclose all conflicts of interest and has mechanisms for the management of such conflicts.

### Access to data

Paper and computer files will be safeguarded from unauthorized access and stored in secure locations. Information collected electronically via the computerized decision-aids will be stored in a secure central location. Information collected on paper forms will be sent to UAB and stored in a secure location. Computer records of study data will be stored in a central database, controlled by a system of user identification and passwords. Patient-physician conversation audio recordings (aim 2) will be kept controlled by unique usernames and passwords, with access to a limited number of study personnel.

Data entered into computerized files will be accessible only to authorized study personnel and will be coded. Touchscreen computers will be password-protected and encrypted by the UAB Informatics, so that in case of a loss of a unit, no patient information can be retrieved.

### Ancillary and post-study care

Study documents will be retained for a minimum of 7 years after the study completion. These documents will be retained for a longer period, however, if required by the IRB or PCORI. No records will be destroyed without prior approval from UAB/CORI.

### Dissemination policy

Dissemination will be ensured with a multi-faceted plan: (1) partner institution/practice leaders have pledged strong support; (2) stakeholder partners from the Arthritis Foundation (AF) and the Lupus Foundation of America (LFA), two key US patient advocacy organizations, will help develop complementary patient messages to drive lupus DA dissemination; (3) institutional resources from three UAB centers, Center for Clinical and Translational Studies (CCTS), Center for Outcomes Effectiveness and Education (COERE), and Comprehensive Arthritis, Musculoskeletal, Bone and Autoimmunity Center (CAMBAC) will allow continuation of technical support to sites for an additional 6 months to facilitate transition to a patient education and QI initiative; (4) study implementation strategies emphasize capacity building in clinics that extend beyond the study period; (5) this study aims to provide a QI package (i.e., a lupus DA implementation manual) which will facilitate spread and sustainability; and (6) ongoing 3-monthly meeting with a multistakeholder panel (patient, clinician, researcher, patient advocacy leaders on stakeholder committee; Additional file 1: Appendix 2) and a steering committee with active engagement on study conduct, troubleshooting, and protocol. A number of the implementation activities emphasize building capacity and overcoming change barriers, which will foster a continuous learning environment [51, 52] where clinics can continue to adapt the existing DA to changing circumstances. Data will be made available to colleagues after obtaining permission from UAB IRB and PCORI.

## Discussion

The purpose of this mixed methods study is to evaluate the impact of different combinations of implementation strategies in promoting the use of an evidence-based decision-aid (DA) for patients with lupus and the conditions that may support or attenuate their effectiveness. The study is significant for a number of reasons. First, lupus is a rare autoimmune disease with significant health outcome disparities, with 2.4 times higher mortality than age-matched controls [15–18]. It affects 0.1% of the population, but lupus nephritis (kidney disease) accounts for 2% of all end-stage renal disease in the USA [1]. Medication decision-making in lupus is challenging [6], with many declining life-saving immunosuppressive medications. The identification of effective strategies for implementing an evidence-based DA can help patients overcome these problems and take advantage of effective treatment options. Second, our study takes a contextually driven approach to assessing implementation strategies that allows participants to choose from a menu of strategies that are believed to be most effective for their unique circumstances (e.g., geography, resources). In doing so, our study explicitly acknowledges that healthcare providers and patients are often situated in unique organizational and community contexts and empirically assesses how these different contexts shape the decisions around the most appropriate strategies for implementing our evidence-based DA [53]. Finally, the study leverages multi-level, multi-method, longitudinal data over a 3-year period from 15 rheumatology clinics from across the USA. These data enable us to examine the study relationships across a variety of contexts and understand how the implementation strategies and their effects on implementation outcomes change over time and why.

The study also faces several challenges. First, despite the inclusion of 15 different clinics from across the USA, our ability to conduct organizational-level analyses will be limited, even with our use of small-N analytic techniques such as fsQCA and qualitative case comparison methods. Likewise, our ability to assess questions of change is predicated on our ability to collect data from the same respondents over time, a non-trivial issue given potential clinical staff and patient turnover [54]. Compounding this second issue is the fact that the study was funded in the pre-COVID-19 pandemic era. We anticipate that unique challenges in patient care, healthcare delivery changes that are impacting outpatient and inpatient care of patients in general, will have both a transient and permanent effect on our study implementation. We are planning to examine this by comparing data in the pre-COVID-19 period to the COVID-19 period. However, these analyses will be limited since the number of patients enrolled during the pandemic is low due to a severe slow down and cessation of patient enrollment during the first 4–6 months of the COVID-19 pandemic in the USA. In response to the COVID-19 pandemic, we modified the patient enrollment with the addition of a virtual patient enrollment in the study for people having their telemedicine clinic visits virtually using telephone or video visits. Patients can now enroll with an e-consent and complete the viewing of the decision-aid and survey virtually, using the links sent to them via email or phone text by using their phone or computer devices. Similarly, the impact of the COVID-19 pandemic on the morale of clinic personnel in general, and specifically towards healthcare quality improvement and patient education efforts, is unknown.

In sum, our study presents a significant opportunity to better understand the most effective strategies for implementing an evidence-based shared decision-aid for patients with lupus and the contextual circumstances that may determine why some strategies are more effective than others. Such insights are especially important given the significant cost and quality consequences of inappropriate treatment for patients with lupus.

## Supplementary Information

**Additional file 1: Appendix 1.** Data Collection and Analyses for Specific Aim 1 and Specific Aim 3. **Appendix 2.** Members of the various IDEAL committees.

## Data Availability

The study PI, biostatistician (Dr. Beasley), and key study investigators (Drs. Hearld, Hall, and Qu) will have access to the final trial dataset. There are no contractual agreements that limit such access for investigators.

## Abbreviations

UAB: University of Alabama at Birmingham
IDEAL: Implementing the DEcision-Aid for Lupus
SDM: Shared decision-making
DA: Decision-aid
ACR: American College of Rheumatology
