# Supplementary material for: Implementing the DEcision-Aid for Lupus (IDEAL): study protocol of a multi-site implementation trial with observational, case study design: Implementing the DEcision-Aid for Lupus
Source: Implement Sci Commun. 2021 Mar 11;2:30. doi: 10.1186/s43058-021-00118-9 (PMC7951119; doi:10.1186/s43058-021-00118-9)
Supplement: Supplementary file 1 — Additional file 1: Appendix 1. Data Collection and Analyses for Specific Aim 1 and Specific Aim 3. Appendix 2. Members of the various IDEAL committees. [file 43058_2021_118_MOESM1_ESM.docx]

**Additional file 1**

**Appendix 1. Data Collection and Analyses for Specific Aim 1 and Specific Aim 3**

|  | | |
| --- | --- | --- |
| *Specific Aim 1 Data Collection and Analyses* | | |
| *Data Type* | *Data Collection Type* | *Analytic Methods* |
| Quantitative | Online & paper survey, Organizational  readiness for implementing change  (ORIC); Team Learning & Psychological  Safety Survey (TLPSS)/ Baseline | Individual & practice level  summary statistics  Correlation |
| Qualitative | Semi‐structured interviews with key  informants/ Baseline | Within‐case/clinic thematic  analysis of CFIR domains  Cross‐case/clinic comparison |
| *Specific Aim 3 Data Collection and Analyses* | | |
| Data Type | Interview Content | Analytic Methods |
| Qualitative | Perceived challenges to long-term  sustained use of the DA  Understand the lessons learned | Within‐case/clinic thematic  analysis of CFIR domains  Cross‐case/clinic comparison |

**Appendix 2. Members of the various IDEAL committees**

The 9-member **stakeholder committee** includes two patients (Leong/Reyes), 2 patient advocacy group leaders (Eakin, Davidson), 2 clinicians (Chatham, Yazdany), 1 DA expert (Fraenkel), 1 linguist (Caro) and 2 researchers (PI, Eisen). Monthly stakeholder committee meetings will address 3 goals: (1) provide potential solutions to problems; and (2) monitor project progress to provide meaningful stakeholder input. The multi-stakeholder platform with differing (sometimes opposing) perspectives will result in lively discussions and thoughtful recommendations. Special consideration will be given patient views, since our objective is to implement a patient-centered DA.

The IDEAL Working Group will meet weekly, identify recruitment challenges early by coordinating with site PIs, and receive guidance from the **Steering Committee** for major study decisions that will meet monthly (Drs. Singh, Saag, Hearld, Hall, Fraenkel, Beasley). Dr. Beasley (**biostatistician**) will chair quarterly meetings of the **scientific expert group** (Singh, co-chair; other members Drs. Hearld, Hall).
